# Clinical and pathologic characteristics of appendiceal neuroendocrine neoplasms diagnosed during pregnancy

**DOI:** 10.3389/fendo.2023.1013638

**Published:** 2023-02-09

**Authors:** Orit Twito, Amit Akirov, Rachel Chava Rosenblum, Dana Herzberg, Kira Oleinikov, Pnina Rotman-Pikielny, Simona Grozinsky-Glasberg

**Affiliations:** ^1^ Endocrine Unit, Wolfson Medical Center, Holon, Israel; ^2^ Sackler Faculty of Medicine, Tel Aviv University, Tel-Aviv, Israel; ^3^ Endocrine Institute, Rabin Medical Center - Beilinson Hospital, Petach-Tikva, Israel; ^4^ Neuroendocrine Tumor Unit, European Neuroendocrine Tumor Society (ENETS) Center of Excellence, Department of Endocrinology, Hadassah Medical Organization and Faculty of Medicine, The Hebrew University of Jerusalem, Jerusalem, Israel; ^5^ Endocrine Institute, Meir Medical Center, Kfar-Saba, Israel

**Keywords:** pregnancy, appendix - appendicitis, neuroendocrine tumor, appendectomy, hemicolectomy

## Abstract

**Introduction:**

Although appendicitis occurs in approximately 1:1000 pregnancies, appendiceal neuroendocrine neoplasm (ANEN) diagnosis during pregnancy is very rare. Data on presentation, treatment and prognosis is scarce.

**Aim:**

To describe ANEN cases diagnosed during pregnancy.

**Materials and methods:**

A retrospective appraisal of 7 consecutive ANEN patients diagnosed during pregnancy from four Israeli tertiary medical centers and comparison with 17 cases described in the literature from 1965-2021.

**Results:**

Age at ANEN diagnosis was 26.4 ± 3.5 years (range 21-33). Patients were diagnosed between gestational weeks 6-40, most frequently in the third trimester (53%). The most common presenting symptom was abdominal pain. Tumor size was 14.3 ± 8.9mm (range 3-45mm). In patients from our series appendiceal base involvement was reported in 2/7; mesoappendiceal invasion in 5/7; lympho-vascular invasion in 2/7. Ki67 staining was reported in 6/7 cases and ranged from 1-10%. Pathology details were lacking in most of the previously published cases. All 7 pregnancies in our series resulted in term delivery with no complications, whereas in historical cases there were one first trimester abortion, one ectopic pregnancy, and one stillbirth. Right hemicolectomy was performed in 5/7 patients in our series and reported in 2/17 historical cases. All hemicolectomies were performed after delivery, 3-16 months after appendectomy. Local metastases were reported in two cases. Follow-up duration was 7-98 months for our patients and 3-48 months in 5 historical cases. No disease recurrence, distant metastases or mortality were noted.

**Conclusions:**

To the best of our knowledge, this is the largest series describing the extremely rare diagnosis of ANEN during pregnancy. Although pathologic characteristics varied, pregnancy outcomes were usually favorable and long-term prognosis was excellent. This data may suggest that a conservative approach to patients with ANEN diagnosis during pregnancy can be considered.

## 1 Introduction

Appendiceal neuroendocrine neoplasm (ANEN) are diagnosed in approximately 0.5% of appendectomies (1–5). These tumors usually harbor excellent prognosis, and rarely require further treatment beyond appendectomy (6, 7). Local lymph node metastases may be present, but distant metastases and disease-related mortality are rare. Right hemicolectomy is suggested for selected patients (8, 9) with large or invasive tumors. Although hormonal hypersecretion syndromes such as carcinoid syndrome and ectopic Cushing’s syndrome have been described in ANEN, these are extremely rare (10, 11).

Although appendicitis and appendectomy occur in approximately 1:1000 pregnancies (12, 13), diagnosis of ANEN during pregnancy is extremely rare. Only 12 cases have been reported in the literature since 1965 (14–23), including only one series of four cases (14). Additionally, five cases of incidental ANEN diagnosed during Cesarian section (CS) have been reported (14, 24, 25).The clinical and pathological data in most of these reports is incomplete.

Epidemiologic and clinical characteristics of ANEN in pregnancy have not been systematically described. The effect of the tumor and its resection on pregnancy outcome is unknown. Moreover, the effect of pregnancy and its unique hormonal and immune milieu on tumor progression and spread has not yet been described. As a result, there are no specific guidelines for evaluation and treatment of patients with ANEN diagnosed during pregnancy. Many clinical issues remain to be resolved, such as sensitivity of imaging studies performed during pregnancy prior to appendectomy; the need for further imaging studies after appendectomy during pregnancy and after delivery; and the sensitivity and specificity of biochemical markers such as chromogranin A and 5-hydroxy-indol-acetic acid (5-HIAA) in this context.

The most important clinical dilemma is patient selection for right hemicolectomy. To date, no pregnancy-specific criteria for right hemicolectomy have been suggested. Furthermore, the preferred timing for hemicolectomy, whether during pregnancy (particularly if ANEN is diagnosed in early pregnancy) or in the post-partum period has not been determined.

In this study, retrospective data were collected from 7 Israeli women with ANEN diagnosed during gestation, as well as 17 cases of gestationally-diagnosed ANEN previously described in the literature. Clinical and pathologic characteristics and long-term follow-up data, where available, are described.

## 2 Materials and methods

Data of 7 ANEN female patients diagnosed during pregnancy were collected from electronic files of four tertiary medical centers in Israel. In addition, data pertaining to another 17 cases described in the literature was retrieved. Data included patient’s age, pregnancy week at diagnosis, presenting symptoms and pre-appendectomy imaging studies. Histopathological characteristics of the tumor included size, location in the appendix, depth of invasion, lympho-vascular invasion (LVI), perineural invasion, immunohistochemical staining and proliferation index (Ki-67). Post appendectomy evaluation included imaging, chromogranin A and 5-HIAA testing; right hemicolectomy indication, timing and outcome (if conducted); pregnancy outcome and long-term surveillance data including tumor recurrence and mortality.

### 2.1 Data analysis

Categorical variables are presented as frequency and percentage. Continuous variables are presented as mean and standard deviation or median and range. Groups were compared using Student t-test. A p-value < 0.005 was considered statistically significant.

### 2.2 Ethical considerations

The study protocol was approved by the Institutional Ethics Committees of the four Medical Centers (Meir Medical Center, Hadassah Medical Center, Rabin Medical Center and Wolfson Medical Center 0143-21-WOMC). In accordance with Helsinki regulations for clinical studies based on chart review, informed consent was waived.

## 3 Results

Data of 19 patients with ANEN diagnosed during pregnancy (7 from our series and 12 previously published in the literature) and 5 ANEN cases diagnosed incidentally at CS (all from previous publications) were included in the analysis (**Table 1**). Historical cases were published between 1965-2019. Patients diagnosed during gestation presented with abdominal pain, and in most cases were suspected to have appendicitis. Patients diagnosed at CS had appendectomy as a routine procedure or due to abnormal appearance of the appendix. There were no cases of pre-operative tumor diagnosis, and no described signs and symptoms of hormone hyper-secretion syndromes.

**Table 1:** Clinical characteristics of 24 appendiceal neuroendocrine neoplasms diagnosed during pregnancy.

Case number	Data source	Age et diagnosis (years)	Pregnancy week	Clinical presentation	Pre-operative imaging	Pregnancy outcome	Follow-up duration (months)
1	Current series	27	27	Abdominal pain	US	NA	98
2	Current series	31	6	Abdominal pain	US	Vaginal term delivery	32
3	Current series	21	38	Abdominal pain	US	Vaginal term delivery	11
4	Current series	26	13	Abdominal pain	US-suspected appendicitis	Vaginal term delivery	23
5	Current series	33	30	Abdominal pain	US	Vaginal term delivery	9
6	Current series	23	22	Abdominal pain	US- appendix not seen, MRI-dilated appendix	CS term delivery	15
7	Current series	24	31	Abdominal pain	us- appendix not seen, MRI-dilated appendix	Vaginal term delivery	7
8	Berrios 1965	23	10	Abdominal pain	NA	Vaginal term delivery	48
9	Berrios 1965	26	12	Abdominal pain	NA	NA	NA
10	Jurica 1989	24	21	Abdominal pain	NA	Stillbirth	NA
11	Mclean 1994	30	37	Abdominal pain	NA	CS term delivery	NA
12	Korkontzelos 2005	23	16	Abdominal pain	NA	CS term delivery	NA
13	Pitiakoudis 2008	24	32	Abdominal pain	NA	Vaginal term delivery	NA
14	Gilboa 2008	31	9	Abdominal pain	Trans vaginal US- edematous appendix	1^st^ trimester abortion	NA
15	Thompson 2011	27	NA	Abdominal pain	NA	Ectopic pregnancy	NA
16	Poiana 2012	27	NA	NA	NA	NA	NA
17	panagiotis 2013	22	27	Abdominal pain	MRI-dilated appendix	NA	NA
18	Piatek 2016	28	25	Abdominal pain	US-appendix not seen	Vaginal term delivery	12
19	Vanags 2017	24	35	Abdominal pain	US-appendix not seen	NA	NA
20	Berrios 1965	21	NA	Routine appendecto-my during CS	irrelevant	irrelevant	NA
21	Berrios 1965	30	38	Routine appendecto-my during CS	irrelevant	irrelevant	NA
22	Syracuse 1979	31	NA	Routine appendecto-my during CS	irrelevant	irrelevant	48
23	Gokaslan 2002	30	NA	Routine appendecto-my during CS	irrelevant	irrelevant	3
24	Janicki 2019	27	40	Routine appendecto-my during CS	irrelevant	irrelevant	36

US, ultrasound; NA, data not available; CS, cesarean section; MRI, magnetic resonance imaging.

**Figure 1:**
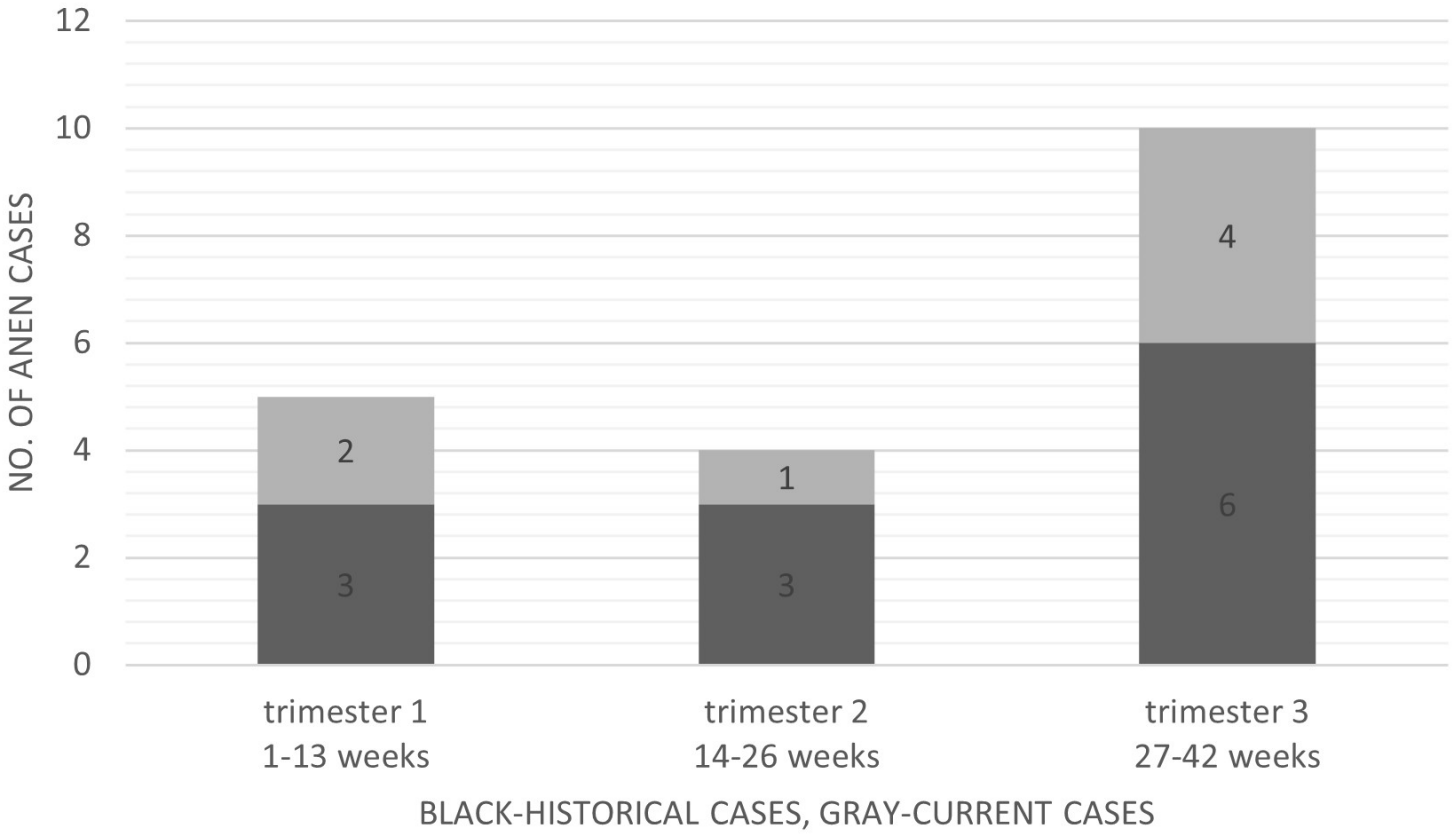
Appendiceal neuroendocrine neoplasms diagnosed during pregnancy divided by pregnancy trimester at diagnosis.

Age at ANEN diagnosis was 26.4 ± 3.5 years (range 21-33); there was no significant age difference between cases diagnosed during pregnancy and those incidentally diagnosed during CS (26 vs. 27.8 years, respectively, p=0.302). As presented at **Figure 1**, patients were diagnosed at gestational week 6-40, but more frequently in the third trimester. In the 7 cases within our series, pregnancy outcomes were favorable (all resulted in term delivery, 6/7 vaginal delivery and 1/7 CS), whereas in historical cases there were one case of first trimester spontaneous abortion five days after appendectomy (26), one case of ectopic pregnancy implanted on the tip of the appendix that was diagnosed during appendectomy (19) and one stillbirth at 21 weeks’ gestation in a patient with concomitant Chlamydia trachomatis infection (15); CS was conducted in 2/5 term deliveries (26). No other post-appendectomy complications were noted.

Data on pre-operative imaging was available for 11 patients. Trans-abdominal ultrasound (US) was performed in in 8/11, magnetic resonance imaging (MRI) in 1/11 and both in 2/11. Trans-abdominal US failed to demonstrate the appendix in 4/11 patients presenting at pregnancy weeks 22-35. MRI demonstrated abnormal findings in the appendix in 3/3 cases. In one additional case, abnormal findings were described in the appendix on vaginal US (26). In all cases the pre-operative imaging results were compatible with the diagnosis of appendicitis but did not reveal the existence of appendiceal tumor.

Tumor histopathological characteristics are presented in **Table 2**. Tumor size was 14.3 ± 8.9 mm (range 3-45 mm). There was no difference between tumors diagnosed during pregnancy and during CS (13.6 vs. 16.4 mm, respectively, p=0.550) or between our series and historical cases (14.8 vs. 13.5 mm, respectively, p=0.574). Other pathology details were incomplete in most of the previously published cases and in one of our cases. Involvement of the appendiceal base was reported in 3/6 of our series and in no historical cases. Mesoappendiceal invasion was reported in 5/6 of our cases and 3 previously reported cases. LVI was reported in 2/7 of our cases and in 2 of previously reported cases. Ki67 staining was reported in 6/7 of our cases and ranged between 1-10%. Only 5/17 historical cases reported Ki67 staining results, which ranged between 1-2%. For one case in the series of Berrios et al, published in 1965, methenoamine silver and ferrous cyanide staining was reported. Neuroendocrine-specific staining (chromogranin A and synaptophysin) was not reported in cases published before 2005. Positive chromogranin staining was reported in 5/7 of our cases and in 6/17 historical cases. Positive synaptophysin staining was reported in 5/7 of our cases and in 4/17 of historical cases.

**Table 2:** Pathology data of 24 Appendiceal Neuroendocrine Neoplasms diagnosed during pregnancy.

Case number	Data source	Tumor size, mm	Tumor location	Mesoappendix invasion	Lympho-vascular invasion	Ki67 (%)	Mitoses/HPF	CgA/synaptophysin staining	inflammation	perforation	Hemicolectomy performed
1	Current series	3	tip	NA	NA	NA	NA	NA/NA	+	–	No
2	Current series	45	base	+	+	1	2	+/+	–	–	Yes, single focus of NEN in fatty tissue
3	Current series	11	tip	+	+	2	NA	+/+	+	–	Yes, no residual tumor
4	Current series	12	base	+	–	4	NA	+/+	+	+	Yes, no residual tumor
5	Current series	13	middle	+	–	10	NA	+/NA	+	–	Yes, no residual tumor
6	Current series	13	NA	+	–	1	0	+/+	–	–	Yes, no residual tumor
7	Current series	14	tip	–	–	2	1	+/+	+	–	No
8	Berrios 1965	3	middle	NA	NA	NA	NA	NA/NA	+	–	NA
9	Berrios 1965	15	middle	NA	NA	NA	NA	NA/NA	NA	NA	NA
10	Jurica 1989	20	tip	–	NA	NA	NA	NA/NA	–	–	NA
11	Mclean 1994	13	NA	+	NA	NA	NA	NA/NA	–	–	No
12	Korkontzelos 2005	22	NA	NA	+	NA	NA	+/NA	–	–	Yes, no residual tumor
13	Pitiakoudis 2008	5	tip	NA	NA	NA	NA	NA/NA	+	–	NA
14	Gilboa 2008	NA	NA	NA	NA	NA	0	+/NA	NA	NA	NA
15	Thompson 2011	NA	NA	NA	NA	NA	NA	NA/NA	NA	NA	NA
16	Poiana 2012	10	Tip	NA	NA	1	NA	+/+	NA	NA	NA
17	panagiotis 2013	4.5	tip	–	NA	2	NA	+/+	+	–	NA
18	Piatek 2016	18	mid	–	NA	1	NA	+/+	+	–	No
19	Vanags 2017	10	NA	NA	NA	1	NA	+/+	NA	NA	NA
20	Berrios 1965	20	NA	NA	NA	NA	NA	NA/NA	NA	NA	NA
21	Berrios 1965	9	Middle	NA	NA	NA	0	NA/NA	NA	NA	NA
22	Syracuse 1979	15	NA	+	NA	NA	NA	NA/NA	–	–	Yes, 2/67 metastatic lymph nodes
23	Gokaslan 2002	20	tip	NA	+	NA	NA	NA/NA	NA	NA	No
24	Janicki 2019	18	NA	+	NA	1	0	+/+	NA	NA	No

CgA, chromogranin A; NA, data not available; NEN, neuroendocrine neoplasm.+ means positive, - means negative, +/+ mean both parameters are positive (CgA and synaptophysin).

Right hemicolectomy was performed in 5/7 patients in our series and reported in 2/17 historical cases. Indications for hemicolectomy were size greater than 2cm in 2 cases, involvement of the appendiceal base in 2 cases, meso-appendiceal invasion in 6 cases, LVI in 3 cases, and Ki67above 2% in 2 cases. All hemicolectomies were performed after delivery, 3-16 months after appendectomy. Hemicolectomy pathology results are presented in **Table 2**. Local metastases were reported in 2 cases: one had a lymph node metastasis, and the other had a focus of tumor in fat tissue.

Follow-up duration was 7-98 months in our series and 3-48 months in five historical cases. No disease recurrence, distant metastases or mortality were noted. All surveillance imaging studies were negative: abdominal US (1 case), abdominal CT (2 cases), abdominal MRI (4 cases), and Ga68-DOTATATE PET-CT (3 cases). Serum chromogranin A testing (5 cases) and urine 5-HIAA testing (5 cases) during follow-up were within normal range.

## 4 Discussion

This is the largest series to date of ANEN diagnosed during pregnancy, incorporating 7 new cases together with a review of 17 historical cases. Treatment of neoplastic disease during pregnancy is challenging due to the inherent dilemma between the desire to protect maternal health and the wish to continue the pregnancy and protect the fetus. This challenge is more pronounced in ANEN as data on tumor behavior during pregnancy is limited, and no international guidelines discuss this rare clinical scenario (8, 9, 27). The aim of this study was to gather existing data on ANEN diagnosed during pregnancy in order to assist in clinical decision making.

### 4.1 Epidemiology

ANEN diagnosis during pregnancy is extremely rare. This is somewhat surprising, because ANEN is more common in women, with a female preponderance of 52-70% of all ANEN patients described in previous reports (2–4, 28, 29). Moreover, ANEN diagnosis is not uncommon in the reproductive years; in a series published by Rosenblum et al, 31% of reported patients were females between 20-40 years of age (1). Suspected appendicitis is the most common non-obstetric indication for surgical intervention during pregnancy. Appendicitis occurs in approximately 1:1000 births (12, 13). Interestingly, the rate of appendicitis is especially low in the third trimester (30, 31), in contrast to the higher rate of ANEN diagnosis in the 3^rd^ trimester observed in this series.

The reasons for the low rates of both appendicitis in the 3^rd^ trimester and ANEN during pregnancy are not well understood. However, clinicians must be alert to this possibility and know to identify relevant symptoms in order to avoid missed diagnosis of ANEN in the appendix.

### 4.2 Presentation

All patients diagnosed during pregnancy in the present cohort were admitted with abdominal pain. The majority had suspected acute appendicitis according to clinical and radiologic parameters. Pre-operative imaging with abdominal US or MRI revealed suspicious features for appendicitis but did not demonstrate the intra-appendiceal tumor. This is not surprising as ANEN are frequently not detected radiologically, most probably due to their small dimensions (32, 33). Since the tumors were not suspected pre-operatively, no patients performed pre-operative biochemical specific testing such as chromogranin A or urine 5-HIAA.

### 4.3 Pathology

Pathology data from previously reported cases was incomplete and did not enable in-depth analysis. Moreover, pathologic processing and diagnosis has changed substantially over the last decades (historical cases were published over seven decades, 1965-2019). Neuroendocrine-specific stainings chromogranin and synaptophysin were not reported in cases published before 2005. Reliable and detailed histopathological data were available in 6/7 of our cohort. Interestingly, a high proportion of tumors in our series had features placing them at ‘high risk’ for persistence/recurrence according to international guidelines. All six patients with ‘high risk’ tumors underwent right hemicolectomy, but residual disease was observed in only one (a patient with a 45 mm tumor involving the appendix base, with invasion of the mesoappendix and blood vessels).

These results highlight the controversy over the indication for right hemicolectomy in patients with ANEN. International guidelines suggest hemicolectomy for ANEN >2 cm or ANEN 1-2 cm with worrisome pathologic features (8, 9). However, some authors have questioned these criteria. For example, a retrospective analysis of 263 ANEN patients found that tumor grade, vascular and lymph vessel invasion were associated with lymph node involvement, while tumor size and mesoappendiceal invasion were not (34). A systematic review including 261 patients from 6 studies found that using a cutoff of 2 cm for hemicolectomy, the number needed to treat was very similar to the number needed to harm (35). Interestingly, a SEER database analysis found that right hemicolectomy gave no survival advantage over appendectomy, even after adjusting for tumor stage and grade (36).

Until large-scale studies are available, the decision whether to perform right hemicolectomy should be made by a NEN expert, within the framework of a multidisciplinary team, and taking the patient’s will into consideration. Posponement of hemicolectomy to the post-partum period seems to be safe, although this series is to small to draw conclusions.

### 4.4 Pregnancy and long term outcomes

In the 7 cases of the current series, no post-operative complications were noted, and pregnancy outcomes were favorable. This is in contrast to prior large series, which described high rates of post-appendectomy complications. For example, in a series of over 7,000 cases, there was an almost two-fold increase of post-appendectomy complications in pregnancy such as sepsis, septic shock, transfusion, pneumonia, bowel obstruction and postoperative infection (13). Moreover, approximately 5% of women experience adverse obstetrical outcomes after appendectomy during pregnancy, especially preterm delivery or miscarriage (37). Wei et al. reported adjusted odds ratios of 1.82 for low birth weight, 1.59 for preterm birth, 1.33 for small for gestational age, 1.24 for CS, and 2.07 congenital anomalies in women with acute appendicitis during pregnancy (38). The discrepancy between our data and data from these large series may be influenced by temporal changes in availability of diagnostic tools, anesthetization and surgery methods. The significance of our data is also limited by the small sample size of our cohort.

Follow-up data was available for all 7 cases of our cohort and only 5 historical cases. No cases of tumor recurrence, distal metastases or mortality were reported. The results of imaging and biochemical studies during follow-up were all negative. These results are in concert with previous studies, and allude to excellent long-term prognosis for ANEN diagnosed during pregnancy (1, 2, 6, 29).

### 4.5 Study limitations

Although this series is the largest reported to date on ANEN diagnosed during pregnancy, its small sample size precludes the formation of definite conclusions.The cases analyzed were treated over a time span of more than 60 years, during which diagnostic and therapeutic approaches have changed substantially. The retrospective nature of the data gives rise to inherent limitations, including potential bias caused by missing or incorrect data.

## 5 Conclusion

ANEN diagnosis during pregnancy is very rare, occurring most commonly during the third trimester. In this series, all cases were diagnosed post-operatively by the pathologist. In most cases, the post-operative period was unremarkable and pregnancy outcomes were favorable. Local metastases were rare and there were no cases of distant metastases or disease related mortality. This data suggests that a conservative approach to patients with ANEN diagnosis during pregnancy may be considered. However, decision-making needs to be individualized and requires discussion within an experienced multidisciplinary team, including a NEN specialist, gynecologist, pathologist and surgeon. The treatment approach should take into consideration not only the risks related to the tumor itself but also the pregnancy-related psychological burden and relevant outcomes. Larger, multi-center studies are warranted to assess the long-term prognosis of this condition, with emphasis on timing and outcomes of both tumor- and pregnancy- related interventions.

## Data availability statement

The raw data supporting the conclusions of this article will be made available by the authors, without undue reservation.

## Ethics statement

The studies involving human participants were reviewed and approved by Edith Wolfson Medical Center Ethics committee. Written informed consent for participation was not required for this study in accordance with the national legislation and the institutional requirements.

## Author contributions

OT conducted the study and wrote the manuscript. AA, RR, DH, KO, PR-P and SG-G conducted the study and revised the manuscript. All authors contributed to the article and approved the submitted version.

## References

[1] Rosenblum RC Klein N Paran H Avital S Kravtsov V Rotman-Pikielny P. Appendiceal tumor incidence and an in-depth look at appendiceal neuroendocrine neoplasm in a cohort of 8,162 appendectomies: Full dataset. Data Brief (2020) 33:106456. doi: 10.1016/j.dib.2020.106456 33225025 PMC7666302

[2] Twito O Paran H Avital S Kravtsov V Rosenblum RC Rotman-Pikielny P. Temporal trends in incidence, evaluation and management of neuroendocrine neoplasms of the appendix: 14 years’ experience. Am J Surg (2021) 221(5):1000–4. doi: 10.1016/j.amjsurg.2020.09.021 33004142

[3] Pawa N Clift AK Osmani H Drymousis P Cichocki A Flora R. Surgical management of patients with neuroendocrine neoplasms of the appendix: Appendectomy or more. Neuroendocrinology (2018) 106(3):242–51. doi: 10.1159/000478742 28641291

[4] Connor SJ Hanna GB Frizelle FA. Appendiceal tumors: Retrospective clinicopathologic analysis of appendiceal tumors from 7,970 appendectomies. Dis Colon Rectum (1998) 41(1):75–80. doi: 10.1007/BF02236899 9510314

[5] Kunduz E Bektasoglu HK Unver N Aydogan C Timocin G Destek S. Analysis of appendiceal neoplasms on 3544 appendectomy specimens for acute appendicitis: retrospective cohort study of a single institution. Med Sci Monit (2018) 24:4421–6. doi: 10.12659/MSM.908032 PMC6053944 29947345

[6] Grozinsky-Glasberg S Alexandraki KI Barak D Doviner V Reissman P Kaltsas GA. Current size criteria for the management of neuroendocrine tumors of the appendix: Are they valid? Clinical experience and review of the literature. Neuroendocrinology (2013) 98(1):31–7. doi: 10.1159/000343801 23051855

[7] Alexandraki KI Kaltsas GA Grozinsky-Glasberg S Chatzellis E Grossman AB. Appendiceal neuroendocrine neoplasms: Diagnosis and management. Endocr Relat Cancer (2016) 23(1):R27–41. doi: 10.1530/ERC-15-0310 26483424

[8] Pape UF Niederle B Costa F Gross D Kelestimur F Kianmanesh R. ENETS consensus guidelines for neuroendocrine neoplasms of the appendix (excluding goblet cell carcinomas). Neuroendocrinology (2016) 103(2):144–52. doi: 10.1159/000443165 26730583

[9] Kunz PL Reidy-Lagunes D Anthony LB Bertino EM Brendtro K Chan JA. Consensus guidelines for the management and treatment of neuroendocrine tumors. Pancreas (2013) 42(4):557–77. doi: 10.1097/MPA.0b013e31828e34a4 PMC4304762 23591432

[10] Grossman AB Kelly P Rockall A Bhattacharya S McNicol A Barwick T. Cushing’s syndrome caused by an occult source: Difficulties in diagnosis and management. Nat Clin Pract Endocrinol Metab (2006) 2(11):642–7. doi: 10.1038/ncpendmet0327 17082811

[11] Diwaker C Shah RK Patil V Jadhav S Lila A Bandgar T. 68Ga-DOTATATE PET/CT of ectopic cushing syndrome due to appendicular carcinoid. Clin Nucl Med (2019) 44(11):881–2. doi: 10.1097/RLU.0000000000002766 31524683

[12] Moltubak E Landerholm K Blomberg M Redéen S Andersson RE. Major variation in the incidence of appendicitis before, during and after pregnancy: A population-based cohort study. World J Surg (2020) 44(8):2601–8. doi: 10.1007/s00268-020-05524-z 32328784

[13] Abbasi N Patenaude V Abenhaim HA. Management and outcomes of acute appendicitis in pregnancy-population-based study of over 7000 cases. BJOG (2014) 121(12):1509–14. doi: 10.1111/1471-0528.12736 24674238

[14] Berrios JR Dunnihoo DR Gibbs CE Moore SF. Appendiceal carcinoid tumors in pregnancy. Obstet Gynecol (1965) 26:428–31. 14341218

[15] Jurica JV Baumgardner DJ. Chlamydia and incidental carcinoid tumor in spontaneous abortion. J Am Board Fam Pract (1989) 2(2):126–9. doi: 10.3122/jabfm.2.2.126 2711879

[16] McLean LK Roussis P Md BB Cox S Miller F. Carcinoid tumors and pregnancy: case report and review of the literature. J Matern Fetal Neonatal Med (1994) 3(3):139–41. doi: 10.3109/14767059409017360

[17] Korkontzelos I Papanicolaou S Tsimoyiannis I Kitsiou E Stefos T Tsanadis G. Large Carcinoid tumor of the appendix during pregnancy. Eur J Obstet Gynecol Reprod Biol (2005) 118(2):255–7. doi: 10.1016/j.ejogrb.2004.07.026 15653215

[18] Pitiakoudis M Kirmanidis M Tsaroucha A Christianakis E Filippou D Sivridis E. Carcinoid tumor of the appendix during pregnancy. A rare case and a review of the literature. J BUON. (2008) 13(2):271–5. 18555477

[19] Thompson RJ Hawe MJG. A rare pathological trinity: An appendiceal ectopic pregnancy, acute appendicitis and a carcinoid tumour. Ir J Med Sci (2011) 180(2):579–80. doi: 10.1007/s11845-009-0283-y 19198975

[20] Poiana C Carsote M Trifanescu R Terzea D Croitoru A. Case study of appendiceal carcinoid during pregnancy. J Med Life (2012) 5(3):325–8. PMC3465003 23049637

[21] Dimitriadis PA Makar RR Kingston G Farouk R. Appendiceal endometriosis and carcinoid presented as acute appendicitis in pregnancy: A rare case report and review of the literature. Case Rep Obstet Gynecol (2013) 2013:360459. doi: 10.1155/2013/360459 23533862 PMC3600218

[22] Piatek S Gajewska M Panek G Wielgos M. Carcionoid of the appendix in pregnant woman - case report and literature review. Neuro Endocrinol Lett (2017) 37(8):535–9. 28326748

[23] Vanags A Strumfa I Simtniece Z Gardovskis J. Neuroendocrine tumour of the appendix in pregnancy. Acta Chirurgica Latviensis (2013) 13(2):81–3. doi: 10.2478/chilat-2014-0017

[24] Syracuse DC Perzin KH Price JB Wiedel PD Mesa-Tejada R. Carcinoid tumors of the appendix. mesoappendiceal extension and nodal metastases. Ann Surg (1979) 190(1):58–63. doi: 10.1097/00000658-197907000-00013 464679 PMC1344458

[25] Gökaslan H Sişmanoğlu A Kaya H Durmuşoğlu F. Incidental carcinoid of appendix in cesarean section. Eur J Obstet Gynecol Reprod Biol (2002) 104(1):76–8. doi: 10.1016/S0301-2115(02)00057-X 12128269

[26] Gilboa Y Fridman E Ofir K Achiron R. Carcinoid tumor of the appendix: Ultrasound findings in early pregnancy. Ultrasound Obstet Gynecol (2008) 31(5):576–8. doi: 10.1002/uog.5313 18393270

[27] Pape U-F Perren A Niederle B Gross D Gress T Costa F. ENETS consensus guidelines for the management of patients with neuroendocrine neoplasms from the jejuno-ileum and the appendix including goblet cell carcinomas. Neuroendocrinology (2012) 95(2):135–56. doi: 10.1159/000335629 22262080

[28] Landry CS Woodall C Scoggins CR McMasters KM Martin RCG. Analysis of 900 appendiceal carcinoid tumors for a proposed predictive staging system. Arch Surg (2008) 143(7):664–70. doi: 10.1001/archsurg.143.7.664 18645109

[29] Shapiro R Eldar S Sadot E Papa MZ Zippel DB. Appendiceal carcinoid at a large tertiary center: Pathologic findings and long-term follow-up evaluation. Am J Surg (2011) 201(6):805–8. doi: 10.1016/j.amjsurg.2010.04.016 21741512

[30] Andersson RE Lambe M. Incidence of appendicitis during pregnancy. Int J Epidemiol (2001) 30(6):1281–5. doi: 10.1093/ije/30.6.1281 11821329

[31] Zingone F Sultan AA Humes DJ West J. Risk of acute appendicitis in and around pregnancy: A population-based cohort study from England. Ann Surg (2015) 261(2):332–7. doi: 10.1097/SLA.0000000000000780 24950289

[32] Pickhardt PJ Levy AD Rohrmann CA Kende AI. Primary neoplasms of the appendix: Radiologic spectrum of disease with pathologic correlation. Radiographics (2003) 23(3):645–62. doi: 10.1148/rg.233025134 12740466

[33] Coursey CA Nelson RC Moreno RD Dodd LG Patel MB Vaslef S. Carcinoid tumors of the appendix: Are these tumors identifiable prospectively on preoperative CT? Am Surg (2010) 76(3):273–5. doi: 10.1177/000313481007600306 20349655

[34] Galanopoulos M McFadyen R Drami I Naik R Evans N Luong TV. Challenging the current risk factors of appendiceal neuroendocrine neoplasms: Can they accurately predict local lymph nodal invasion? Results from a Large case series. Neuroendocrinology (2019) 109(2):179–86. doi: 10.1159/000499381 31060039

[35] Ricci C Ingaldi C Alberici L Brighi N Santini D Mosconi C. Histopathological diagnosis of appendiceal neuroendocrine neoplasms: When to perform a right hemicolectomy? A systematic review and meta-analysis. Endocrine (2019) 66(3):460–66. doi: 10.1007/s12020-019-01984-z 31227991

[36] Guzman C Boddhula S Panneerselvam N Dodhia C Hellenthal NJ Monie D. Appendiceal carcinoid tumors: Is there a survival advantage to colectomy over appendectomy? J Gastrointest Surg (2019) 24(5):1149–57. doi: 10.1007/s11605-019-04306-w 31273553

[37] Sachs A Guglielminotti J Miller R Landau R Smiley R Li G. Risk factors and risk stratification for adverse obstetrical outcomes after appendectomy or cholecystectomy during pregnancy. JAMA Surg (2017) 152(5):436–41. doi: 10.1001/jamasurg.2016.5045 PMC5831452 28114513

[38] Wei P-L Keller JJ Liang H-H Lin H-C. Acute appendicitis and adverse pregnancy outcomes: A nationwide population-based study. J Gastrointest Surg (2012) 16(6):1204–11. doi: 10.1007/s11605-012-1858-x 22402956

